# Microbial Corrosion Behavior of L245 Pipeline Steel in the Presence of Iron-Oxidizing Bacteria and *Shewanella algae*

**DOI:** 10.3390/microorganisms13071476

**Published:** 2025-06-25

**Authors:** Fanghui Zhu, Yiyang Liu, Chunsheng Wu, Kai Li, Yingshuai Hu, Wei Liu, Shuzhen Yu, Mingxing Li, Xiaohuan Dong, Haobo Yu

**Affiliations:** 1 Oil & Gas Technology Research Institute of ChangQing Oilfield Company, Xi’an 710018, China; 2 Beijing Key Laboratory of Failure, Corrosion and Protection of Oil/Gas Facility Materials, College of New Energy and Materials, China University of Petroleum-Beijing, 18 Fuxue Road, Changping, Beijing 102249, China

**Keywords:** microbial corrosion, iron oxidizing bacteria, *Shewanella algae*, symbiotic corrosion

## Abstract

Microbiologically influenced corrosion (MIC) poses significant challenges in oilfield water injection environments, leading to substantial socioeconomic losses. L245 steel, a low-alloy steel widely used in oil and gas pipelines due to its excellent mechanical properties and cost-effectiveness, remains highly vulnerable to MIC during long-term service. This study uses surface characterization and electrochemical techniques to investigate the corrosion behavior of L245 pipeline steel under short-cycle conditions in a symbiotic environment of iron-oxidizing bacteria (IOB) and *Shewanella algae* (*S. algae*). Key findings revealed that localized corrosion of L245 steel was markedly exacerbated under coexisting IOB and *S. algae* conditions compared to monoculture systems. However, the uniform corrosion rate under symbiosis fell between the rates observed in the individual IOB and *S. algae* systems. Mechanistically, the enhanced corrosion under symbiotic conditions was attributed to the synergistic electron transfer interaction: IOB exploited electron carriers secreted by *S. algae* during extracellular electron transfer (EET), which amplified the microbial consortium’s capacity to harvest electrons from the steel substrate. These results emphasize the critical role of interspecies electron exchange in accelerating localized degradation of carbon steel under complex microbial consortia, with implications for developing targeted mitigation strategies in industrial pipelines exposed to similar microbiological environments.

## 1. Introduction

In recent years, with the further development of natural resources, many metal materials have faced the risk of corrosion failure caused by environmental microorganisms in the process of use. The phenomenon of microbial corrosion (MIC) is widely found in the natural environment, such as the ocean and soil, as well as in the industrial environment, such as petroleum and aerospace [1,2]. According to statistics, 50% of the causes of oil and gas pipeline leakage include the influence of microorganisms on corrosion [3].

Microbiologically influenced corrosion (MIC) exhibits complex mechanisms. Since early observations correlating microbial activity with metal corrosion last century [4], MIC has been defined as corrosion directly or indirectly induced by microbial metabolic processes and associated metabolites [5]. In addition, microorganisms will form a layer of extracellular polymers composed of microbial cells and metabolites on the material’s surface. Their uneven and random coverage characteristics will cause the concentration cell phenomenon of oxygen and other ions in different areas of the material surface. Therefore, some scholars have proposed the mechanism of corrosion concentration cells [6]. In recent years, with the development of research in microbial fuel cells, it has been found that the electron transfer phenomenon in the life metabolic activities of some corrosive microorganisms can cause electron transfer between microbial cells and metal material surfaces. Researchers believe this is a more common mechanism for microorganisms to cause metal corrosion [7,8]. With the introduction of these new mechanisms, the corrosion mechanism of most microorganisms on materials has also been well explained.

*Shewanella algae* (*S. algae*) is a Gram-negative, facultatively anaerobic bacterium. Its cells are generally rod-shaped and widely present in the marine environment. It is a typical electroactive microorganism [9]. *Shewanella* have been isolated from natural environments with different temperatures, osmotic pressures, and salt concentrations, from which they can be found to be highly viable [10,11,12]. Many previous studies have confirmed that *Shewanella* can accelerate the corrosion of stainless steel and titanium alloys [13,14], and the extracellular electron transfer between metal and metal plays a vital role in accelerating the process of microbial corrosion [15]. However, it has also been reported that *Shewanella* can inhibit the pitting corrosion of stainless steel [16,17], so it is of great significance to study the effect of *Shewanella* on pipeline corrosion. Iron-oxidizing bacteria (IOB) are one of the most abundant and destructive aerobic bacteria in carbon steel pipelines [18]. As an electron acceptor, oxygen makes IOB generate energy for growth by oxidizing Fe^2+^ to Fe^3+^, producing oxygen concentration cells on the metal surface, accelerating metal dissolution, and causing corrosion [19,20]. *Shewanella* and iron-oxidizing bacteria are widely distributed in different environments due to their metabolic diversity and adaptability [21], and the colonies in the microbial membrane are symbiotic. There may be complex interactions between them, which make the microbial corrosion mechanism unclear [22,23]. Currently, most of the related research work adopts single-bacteria environment experiments, and there is a lack of research on the corrosion behaviors and laws under the coexistence conditions of *Shewanella* and iron-oxidizing bacteria, which is closer to the actual working condition environment. Therefore, it is of great significance to investigate the corrosion behavior of microorganisms under the coexistence of *Shewanella* and IOB for the long-term safe operation of pipelines.

In this paper, the pitting pits under the film were statistically analyzed using SEM observation of biofilm and corrosion morphology, the weight loss method to calculate uniform corrosion rate, and a laser confocal microscope to measure pitting depth. The corrosion behavior of L245 pipeline steel in the presence of iron-oxidizing bacteria and *Shewanella* and its role in the MIC process under their symbiotic conditions were investigated by open circuit potential measurement and electrochemical impedance spectroscopy.

## 2. Materials and Methods

### 2.1. Materials

#### 2.1.1. Experiment Reagent

In this study, IOB (Iron-oxidizing bacteria) were provided by a water injection well in an oil field and cultured in an anaerobic bottle with culture medium. The medium composition is shown in Table 1. The strain of *S. algae* was isolated from the purchased finished 2216E seawater medium, with the medium composition shown in Table 2. The pH of the medium was adjusted to 7.0–7.2, after which the medium was autoclaved at 121 °C for 20 min for both microorganisms. The medium was also deoxygenated by passing nitrogen for 1 h per liter for 2 h after sterilization.

#### 2.1.2. Testing Material

In this study, the hanging piece of L245 material was selected as the experimental material, with a size of 30 × 10 × 5 mm, and its composition is shown in Table 3. Before experiments, all the samples were ground in sequence with 100#, 400#, 800#, 1000#, and 1200# grit sandpapers in ascending order and rinsed with deionized water and ethanol. The samples were then stored in a desiccator. Before use, each specimen was disinfected by exposure to an ultraviolet lamp for one hour.

### 2.2. Methods

#### 2.2.1. Corrosion Experiments

Add 5 mL of bacterial solution and 200 mL of the corresponding medium to the 250 mL anaerobic bottle and three prepared L245 hanging pieces. In the symbiotic experiment of the two bacteria, 3 mL of each bacterium and 100 mL of the corresponding medium were added. Then, the anaerobic bottle was placed in a constant temperature incubator for 7 days, and the culture temperature was 38 °C.

After 7 days of static corrosion test in the culture medium containing *S. algae*, IOB, and mixed strains, the samples were washed three times with sterile phosphate-buffered saline (PBS) solution to remove the dead and loosely attached bacteria. Before using scanning electron microscopy to characterize the surface biofilm morphology, the samples were fixed and then dehydrated with different concentrations of ethanol (25%, 50%, 75%, and 100%), each for 10 min, except for the final step, for 30 min.

#### 2.2.2. Corrosion Rate Calculation

Before soaking, the original weight of the sample was measured using an electronic balance. After the immersion test, the corroded samples were taken out. Before calculating the corrosion rate, the biofilm and product film on the sample’s surface need to be cleaned with a pickling solution and then washed with deionized water and anhydrous ethanol. The specimen uniform corrosion rate formula is as follows:

(1) $$Ra=\frac{365\times 24\times (W1-W2)}{\rho At}$$

where W1 and W2 are the front weight and back weight of the sample, respectively, and the unit is g; for example, the density of L245 is 0.00785 g/mm^3^. A is the sample’s surface area, and the unit is mm^2^; t is the experimental period in hours. The above units are set to be practical measurement units without further conversion, and the calculated uniform corrosion rate unit is millimeters per year, that is, mm/a.

#### 2.2.3. Corrosion Sample Morphology Observation

After 7 days of corrosion test, the biofilm and corrosion products on the surface of the hanging sheet were removed by pickling solution, and the sample was bonded to the sample table with conductive adhesive. The surface corrosion morphology of the corrosion test after cleaning was observed by scanning electron microscope (SEM). The depth and morphology of pitting on the sample’s surface washed clean with pickling solution were measured by laser confocal microscopy (CLSM), and the pitting rate was calculated according to the maximum pitting depth on the sample’s surface in each medium solution.

#### 2.2.4. Electrochemical Measurements

Electrochemical tests were carried out by an electrochemical workstation with a three-electrode cell, in which the steel, platinum plate, and saturated calomel electrode (SCE) served as the working electrode (WE), counter electrode (CE), and reference electrode (RE), respectively. First, the L245 hanging piece is cut into a 10 mm × 10 mm × 5 mm sample, a 10 mm × 10 mm square surface is used as the bare surface of the electrode, and the copper wire is welded on the back. The acrylic powder is mixed with the curing agent and sealed after mixing, and the electrode is poured into the mold. After curing successfully, use 800#, 1000#, and 1200# sandpaper to grind, clean with anhydrous ethanol, blow dry, and finally fix with other electrodes in a five-port bottle sealed preservation. In the experiment of *Shewanella* and iron-oxidizing bacteria, 500 mL of medium and 10 mL of bacterial solution were added. In the symbiotic experiment of the two bacteria, 250 mL and 50 mL bacterial solutions of each bacterial medium were added, respectively.

The bottles were placed in a thermostat with an incubation temperature of 38 °C, and their open-circuit potentials were measured daily. Measuring the open circuit potential (OCP) for 10 min to ensure the stability of the electrochemical system. Electrochemical impedance spectroscopy (EIS) was tested by applying a sinusoidal perturbation of 10 mV and a frequency range of 10^5^ Hz to 10^−2^ Hz. Appropriate equivalent circuit analysis of EIS data was performed using ZSimpWin software 3.6. On the last day, the polarization curve was measured after the open-circuit potential and impedance measurements were completed. The polarization curve test was performed within ±0.5 V of the stable OCP value, and the scanning rate was 1 mV/s.

### 2.3. Experimental Equipment

The experimental instruments in this paper are shown in Table 4.

## 3. Results

### 3.1. Maximum Uniform Corrosion Rate and Pitting Depth

**Figure 1:**
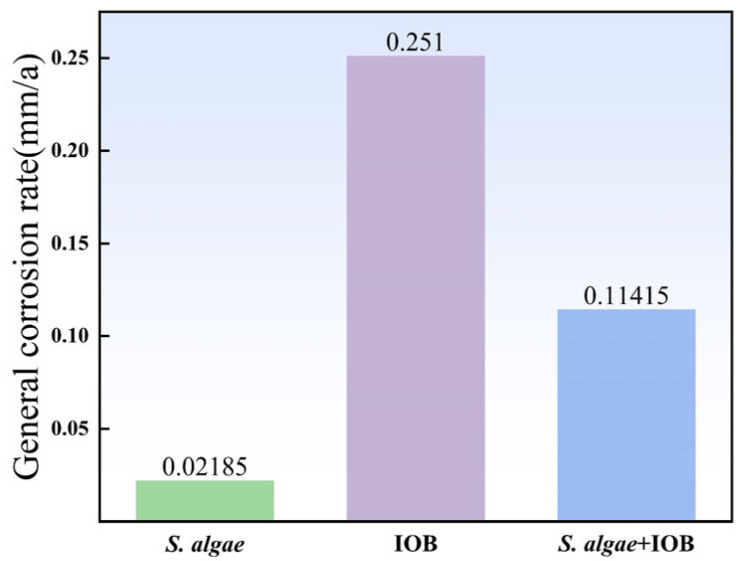
Uniform corrosion rate.

Figure 1 shows the corrosion rates of the samples after 7 days of corrosion at 38 °C in single bacterial and symbiotic media. For the three corrosion conditions, the uniform corrosion rate in the IOB medium reached 0.25 mm/a, and the corrosion rate of the sample in the *S. algae* medium was only 0.02 mm/a. The corrosion rate in the IOB medium alone was 10 times higher than in the *S. algae* solution. The difference indicates that IOB causes more serious corrosion damage than *S. algae*. In the mixed medium, the corrosion rate after symbiosis is between the single IOB and *S. algae* medium, and its uniform corrosion rate is 0.11 mm/a.

**Figure 2:**
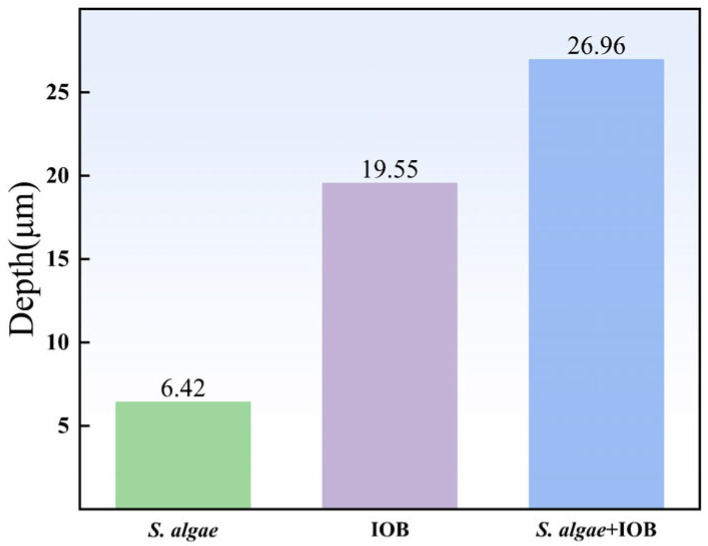
Maximum pit depth.

Figure 2 shows the maximum pitting depth statistics for the three conditions. The maximum pitting depths of *S. algae*, IOB, and mixed strains were 6.42 μm, 19.55 μm, and 26.96 μm, respectively. Although the uniform corrosion rates showed the order of influence as *S. algae* < mixed strains < IOB, the data of pitting corrosion did not show this trend, which also reflects that the changing trend of localized corrosion and uniform corrosion is not necessarily the same.

### 3.2. Cell Count Measurements

**Figure 3:**
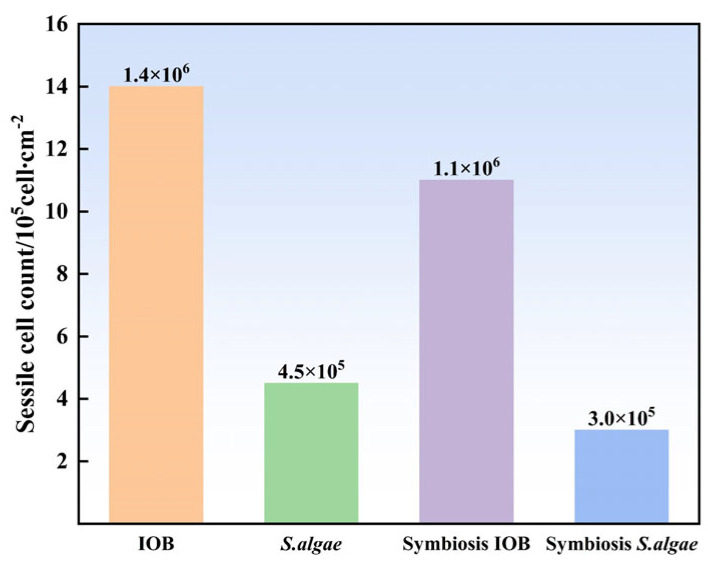
Number of sessile cells on the surfaces of L245 pipeline steel.

The differences in the number of sessile microbes on the samples immersed in the different systems were investigated using MPN. After 7 days of cultivation, the number of sessile microbes in 1 cm^2^ of corrosion products of L245 steel samples is shown in Figure 3. Extracellular electron transfer in biofilms is a key factor in the microbial corrosion of carbon steel, so the number of sessile cells on the carbon steel surface has a significant impact on its corrosion. As shown in the figure, the number of sessile microbes on the IOB sample was 1.4 × 10^6^ cells/cm^2^, while the number of sessile microbes on the *S. alage* sample was 4.5 × 10^5^ cells/cm^2^. The number of sessile microbes under mixed medium was lower than that in the single system. This may be attributed to nutritional limitations hindering microbial reproduction, leading to competition between bacteria and subsequently inhibiting their metabolic activities.

### 3.3. Surface Morphology Observation

**Figure 4:**
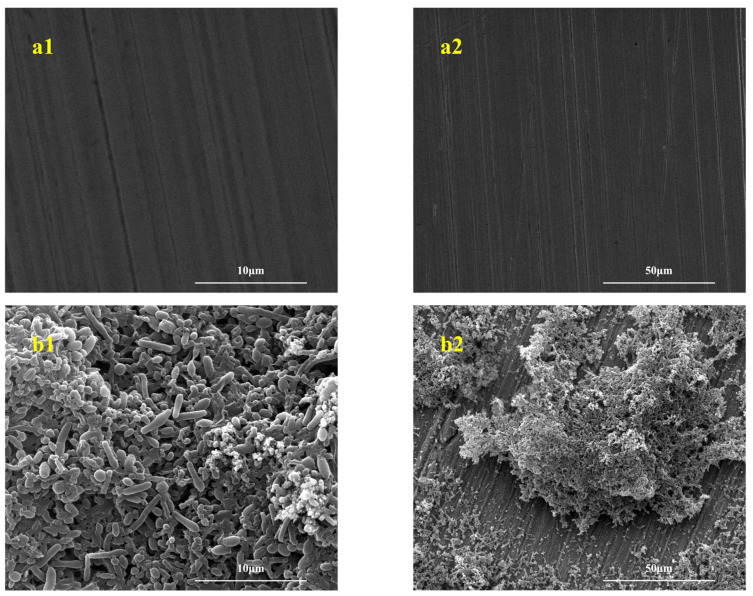

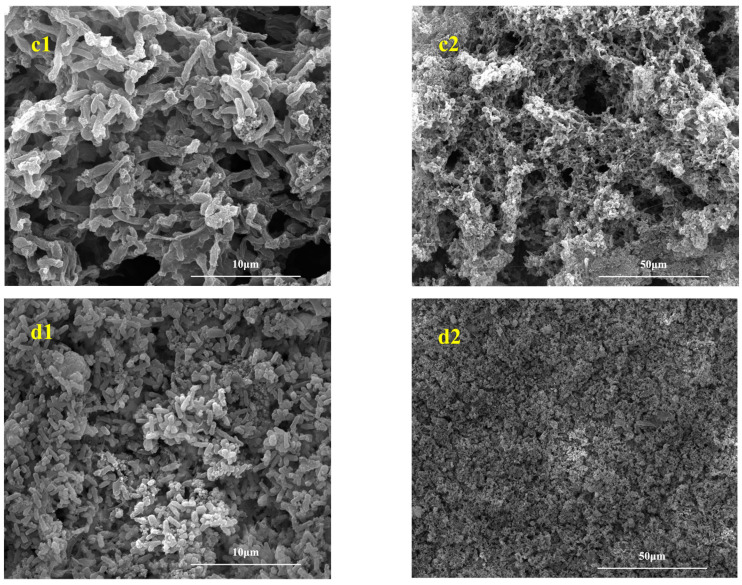
SEM images of biofilm. (**a1**,**a2**) *blank control*; (**b1**,**b2**) *S. algae*; (**c1**,**c2**) IOB; (**d1**,**d2**) *S. algae* + IOB.

Figure 4 shows the SEM images of biofilms taken by the blank control group, IOB, *S. algae*, and mixed strains symbiosis experiments. As shown in the figure, after a 7-day immersion test, the surface of the sterile control group steel substrate showed no obvious corrosion, no biofilm formation, and clear processing marks on the sample, indicating that microorganisms caused the corrosion. In the experiments with *S. algae* alone, the substrate surface formed only incomplete biofilms with sparse coverage, and a large number of bacteria could be directly observed in the biofilm-covered areas. In the single IOB system experiment, the biofilm growth was relatively dense and complete, and a large number of bacteria could be directly observed on the biofilm. In the mixed-strain symbiotic system, a complete and dense biofilm formed on the surface of the L245 suspension plate, with higher maturity and activity than the biofilm in the IOB system alone experiment.

**Figure 5:**
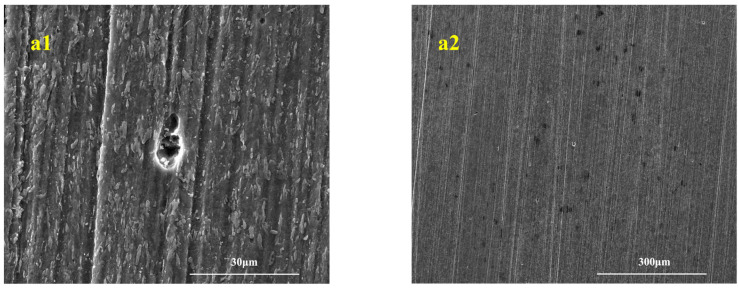

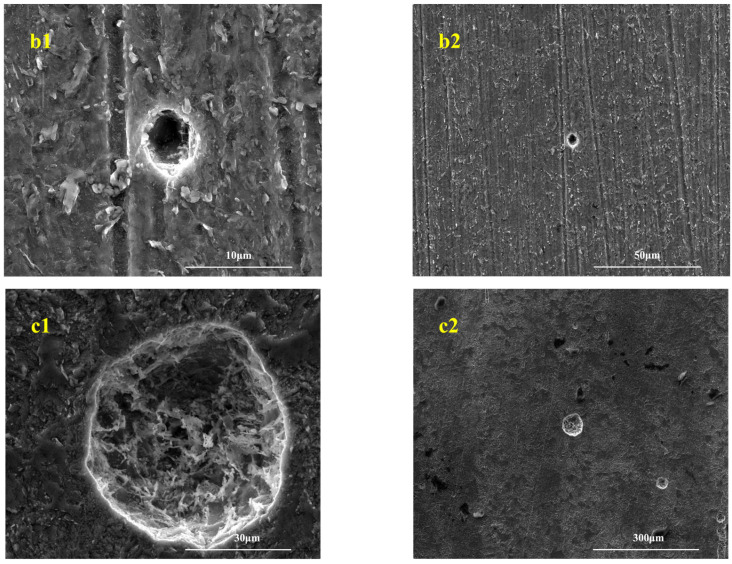
Pitting SEM images. (**a1**,**a2**) *S. algae*; (**b1**,**b2**) IOB; (**c1**,**c2**) *S. algae* + IOB.

After removing the biofilm, confocal laser scanning microscopy (CLSM) was used to analyze the surface in more detail, as shown in Figure 5. Under the three experimental conditions, the samples exhibited obvious pitting corrosion. Among them, the pitting size of the sample in the mixed-strain symbiotic medium was larger than that of the other two groups, and it was difficult to see polishing traces. Polishing traces can be seen on the sample’s surface in the *S. algae* and IOB single system medium, with the most obvious traces observed in the *S. algae* medium, where only slight corrosion and pitting occurred.

**Figure 6:**
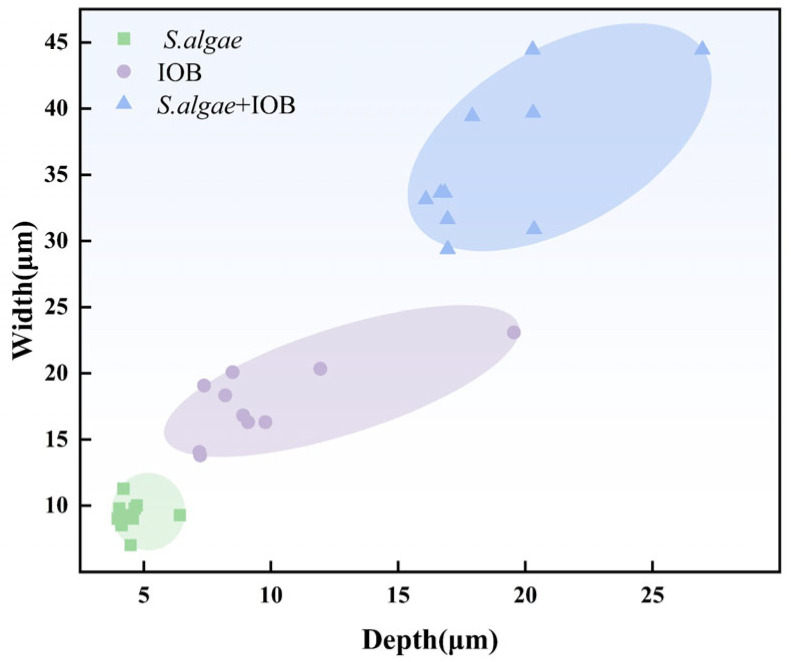
Scatter plot of pitting data of *S. algae* and the IOB symbiosis experiment.

Figure 6 is a scatter plot of pitting data collected from test specimens under three experimental conditions: IOB, *S. algae*, and mixed strains. Under the single IOB condition, the depth and width of pitting corrosion were higher than those of *S. algae* single bacteria. In contrast, under the mixed strain symbiotic condition, the depth and width of pitting were significantly higher than those of either *S. algae* or IOB alone. This trend aligns with the maximum pitting depth data: *S. algae* < IOB < mixed strain. This indicates that, under symbiotic conditions, the corrosion ability of mixed colonies is significantly enhanced compared to that of single bacteria. The corrosion ability of single bacteria is influenced by mixed colonies and shows an upward trend, which is consistent with the conclusions obtained from the SEM above.

### 3.4. EIS Measurements

#### 3.4.1. Open Circuit Potential

**Figure 7:**
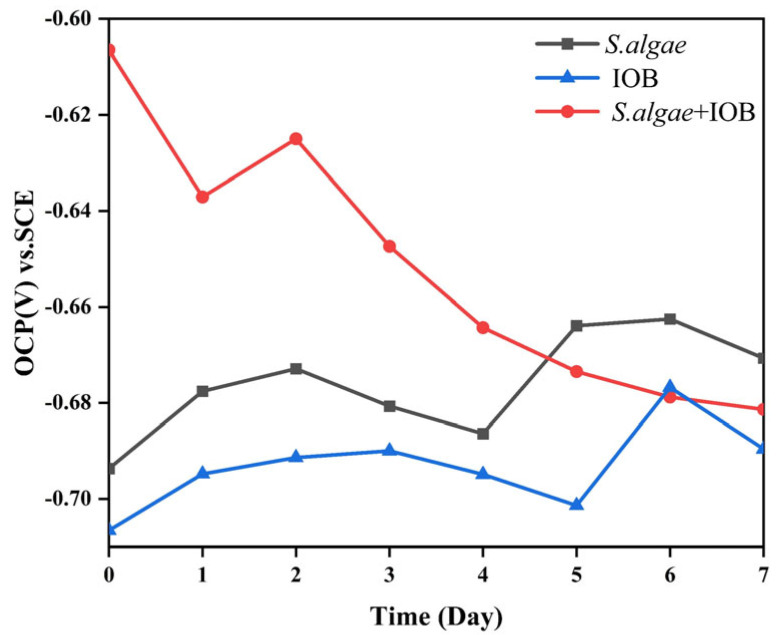
Open circuit potential.

Monitoring of OCP provided some information on the evolution of corrosion. Figure 7 shows the variations of OCP in different media with time. The OCP of the symbiotic system showed a continuous downward trend, starting at −0.61 V. By the end of the seventh day of testing, it had been reduced to −0.68 V, indicating that the corrosion tendency of the symbiotic system was increasing. Notably, the OCP of L245 in medium containing *S. algae* and IOB showed the same trend, which decreased slowly on the 3rd and 2nd days, respectively. They both reached the peak value on the 6th day but decreased on the 7th day, from the initial −0.69 V and −0.71 V increased to −0.67 V and −0.69 V, indicating that the corrosion trends of both systems were decreasing. The OCP values were most negative in the IOB group, followed by *S. algae*, while the OCP value of *S. algae* and IOB symbiosis was the most positive. The greater the potential negative corrosion tendency, the more likely carbon steel corrosion is to occur under the IOB system medium. The order of corrosion tendency was IOB > *S. algae* > mixed strains, which was inconsistent with the uniform corrosion rate.

#### 3.4.2. Polarization Measurements

**Figure 8:**
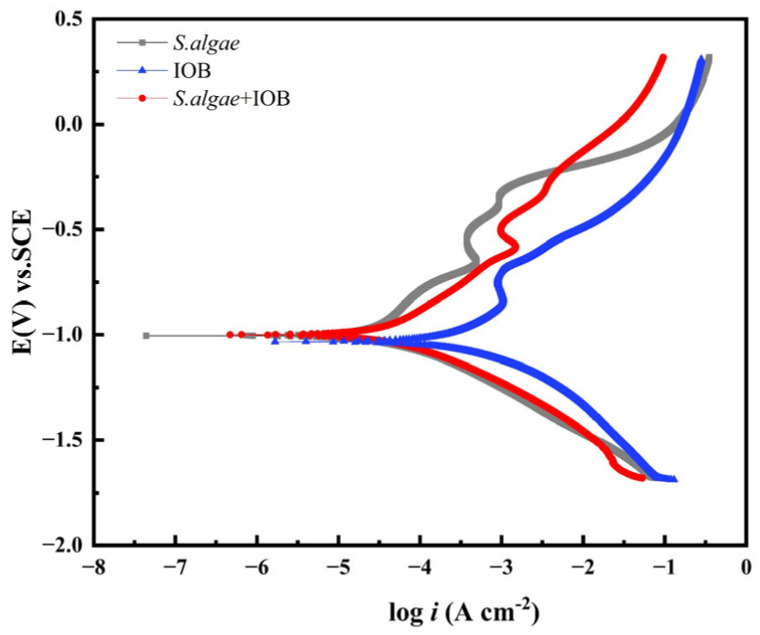
The polarization curves of L245 corroded for 7 days in different microbial systems.

Figure 8 shows the potentiodynamic polarization curves of L245 immersed in different media for 7 days. Table 5 summarizes the corresponding fitted electrochemical parameters, including corrosion potentials (E_corr_) and corrosion current density (I_corr_). The anodic curve showed that the samples were passivated in the culture medium. Compared with the addition of *S. algae* medium alone, the corrosion current density of L245 steel becomes smaller, and the corrosion potential moves to negative in the symbiotic environment of mixed strains, indicating that the corrosion of carbon steel is aggravated in the symbiotic environment. The comparison of corrosion current density is IOB > mixed strain > *S. algae*. A smaller corrosion current density indicates better corrosion resistance, indicating that the corrosion tendency of the IOB group is the largest, which is consistent with the results of a uniform corrosion rate.

#### 3.4.3. Polarization Curve Diagram

**Figure 9:**
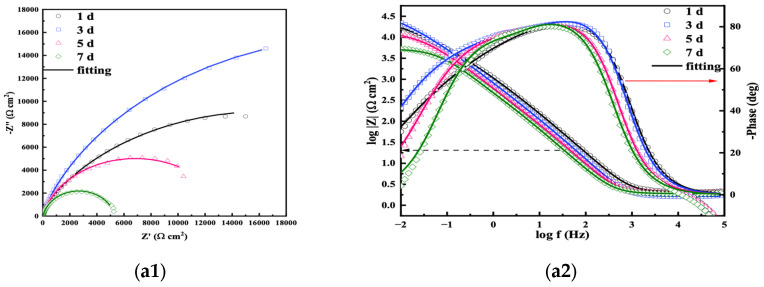

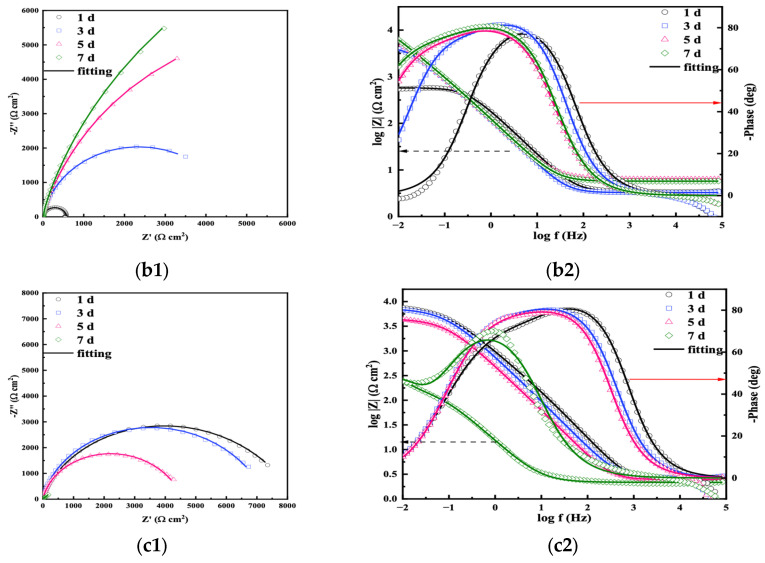
The fitted Nyquist and Bode plots. (**a1**,**a2**) *S. algae*; (**b1**,**b2**) *S. algae* + IOB; (**c1**,**c2**) IOB.

The fitting results are shown in Figure 9, where in the Nyquist diagram of the *S. algae* group, the curve radius increases from day 1 to day 4 and gradually decreases from day 5 to day 7. In general, the size of the impedance arc is related to the formation of corrosion product film on the electrode surface. The denser the corrosion product film is, the more protective the effect on the electrode is. Therefore, a larger impedance arc indicates that the formed film has defects or has been damaged [24,25]. The impedance arc became larger in the first 4 days, forming a dense corrosion product film. Then, the impedance arc gradually decreased, meaning the film was destroyed or peeled off. This reflected that its corrosion resistance initially increased and then continued to decrease after 4 days. In the Nyquist diagram of the mixed strains group, the radius of the curve continued to increase, and its corrosion resistance also continued to increase. The radius of the curve in the IOB group gradually decreased, and the corrosion trend continued to increase.

**Figure 10:**
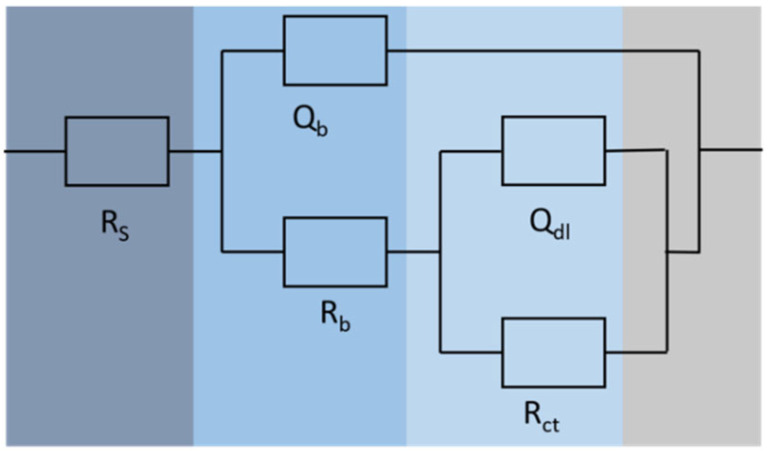
Circuit diagram.

The equivalent circuits (Figure 10) were used to fit the impedance spectrum to further analyze the changes in electrochemical behavior on the surface of L245 immersed in different systems. In the fitting circuit, R_S_ represents the solution resistance, Q_dl_ represents the double-layer capacitance of the corrosion product film, and R_ct_ represents the charge transfer resistance; Q_b_ represents the biomembrane capacitance, and R_b_ represents the biomembrane resistance. The fitting results are shown in Table 6. The R_ct_ value of the *S. algae* is the largest, followed by the mixed strains symbiosis, and the IOB group is the smallest. The R_ct_ is inversely proportional to the corrosion rate, indicating that the IOB group has the largest corrosion tendency, and the results are consistent with the uniform corrosion rate.

## 4. Discussion

During the IOB-driven corrosion process, anodic and cathodic reactions occur on the surface of L245 steel. Metallic iron (Fe^0^) is oxidized to Fe^2+^, which is further accelerated by IOB to Fe^3+^ to obtain energy for bacterial metabolism. The primary electrochemical corrosion reactions induced by IOB on carbon steel are as follows [26].

Anodic reactions:

Fe→Fe^2+^ + 2e^−^, (2)

Fe^2+^→Fe^3+^ + e^−^, (3)

Cathodic reaction (oxygen depolarization):

1/2O_2_ + H_2_O + 2e^−^→2OH^−^, (4)

**Figure 11:**
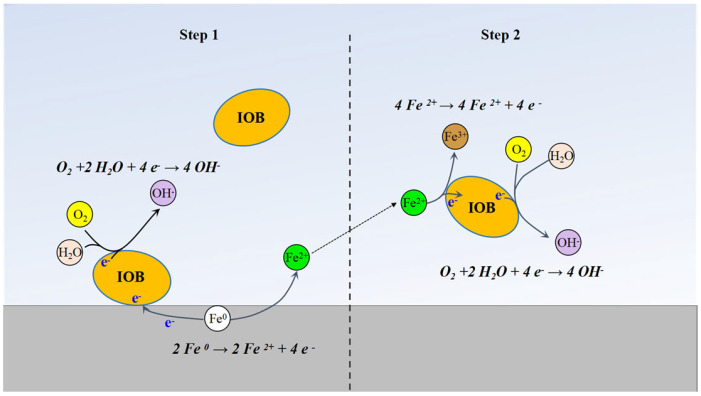
Corrosion mechanism diagram of L245 in IOB.

The generated OH^−^ ions interact with Fe^2+^ and Fe^3+^, forming Fe(OH)_2_ and Fe(OH)_3_. Subsequently, Fe(OH)_2_ is oxidized to FeOOH or transformed into Fe_3_O_4_. FeOOH and Fe(OH)_3_ are unstable and decompose into stable Fe_2_O_3_ [27]. The corrosion mechanism of L245 in IOB is shown in Figure 11.

Simultaneously, Shewanella algae (*S. algae*), as an electroactive bacterium, mediates extracellular electron transfer (EET) to acquire electrons from the iron substrate [28]. Specifically, *S. algae* secretes flavins to shift from oxidized (Rfox) to reduced states (Rfred) during electron uptake [29]. The reduced flavins diffuse toward IOB, enabling the symbiotic colony to utilize electron carriers generated by *S. algae*. This cooperative electron acquisition significantly enhances localized pitting corrosion on L245 steel. Moreover, *S. algae*, functioning as a typical iron-reducing bacterium (IRB) [30], reduces protective Fe^3+^ -containing corrosion products (e.g., Fe_2_O_3_) back to Fe^2+^, which is reoxidized by IOB. This cyclic interplay disrupts passivation layers and exacerbates pitting corrosion.

**Figure 12:**
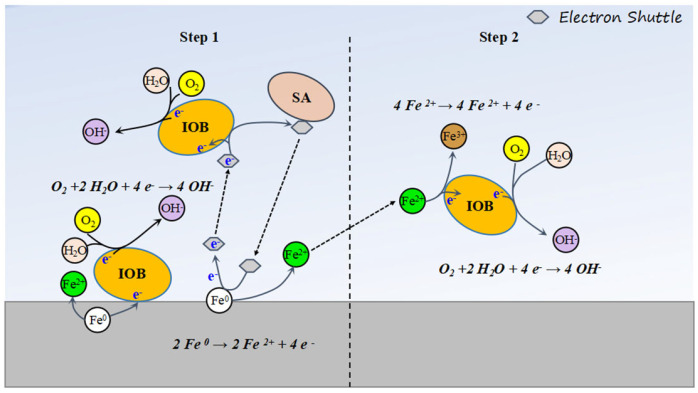
Corrosion mechanism diagram of L245 in mixed media of IOB and *S. algae*.

Notably, under mixed-strain symbiotic conditions, the overall corrosion rate of L245 steel decreases compared to IOB-dominated systems. However, the maximum pit depth increases. This phenomenon arises from nutrient depletion over time, triggering competition between bacterial species and suppressing their metabolic activity, thereby alleviating general corrosion. Meanwhile, the accumulated localized electron transfer and Fe^3+^ reduction/oxidation cycles progressively deepen pits, leading to severe localized degradation. The corrosion mechanism of L245 in IOB and *S. algae* mixed media is shown in Figure 12.

## 5. Conclusions

In this paper, the iron-oxidizing bacteria enriched and cultured in the field water were separately inoculated and co-cultured with *S. algae*. Combined with surface morphology observation, corrosion product analysis, and electrochemical test, the synergistic effect of microorganisms on the corrosion process of L245 steel was revealed. The results show that compared with *S. algae* alone, the pitting corrosion of L245 steel in the symbiotic environment of mixed strains is aggravated, the corrosion rate is increased to 0.11 mm/a, the corrosion current density is reduced, and the corrosion potential is shifted to negative. The impedance in the symbiotic environment of mixed strains decreases compared with that under *S. algae*, and the corrosion tendency increases. Surface analysis and electrochemical measurements show that the symbiotic environment of mixed strains promotes the corrosion of L245 steel. IOB can use the electron carrier produced by *S. algae*, the symbiotic colony enhances the electron acquisition ability of the matrix metal, and the corrosion ability is better than that of single bacteria.

## Figures and Tables

**Table 1:** Composition of IOB medium.

Reagents	Content (g/L)
Sodium sulfate	0.50
Calcium chloride hexahydrate	0.20
Dipotassium hydrogen phosphate	0.50
Magnesium sulfate heptahydrate	0.50
Sodium nitrate	0.50
Iron ammonium citrate	6.00

**Table 2:** Components of 2216E seawater medium.

Reagents	Content (g/L)
Peptone	5.00
Sodium chloride	19.45
Magnesium chloride	5.98
Sodium sulfate	3.24
Yeast extract powder	1.00
Iron citrate	0.10
Calcium chloride	1.80
Potassium chloride	0.55
Sodium carbonate	0.16
Potassium bromide	0.08
Strontium chloride	0.034
Boric acid	0.022
Sodium silicate	0.004
Sodium fluoride	0.0024
Sodium nitrate	0.0016
Disodium hydrogen phosphate	0.0008
Sodium chloride	19.45

**Table 3:** Chemical composition (mass fraction%) of L245 tubing steel.

Element	Title 2 Content
C	0.18
Mn	0.38
P	0.014
S	0.0086
Si	0.19
Gr	0.04
Ni	0.019
Fe	margin

**Table 4:** Models of experimental instruments and equipment.

Name of Instrument	Model Number
Field emission scanning electron microscope	Quanta 200F
Confocal laser scanning microscopy	OLS5100
Electrochemical workstation	CHI1660E
Biochemical incubator	LRH-250
Electronic balance	ME 104E/02
Autoclave	DGL

**Table 5:** Polarization curve fitting parameters.

Experimental Condition	β_a_ (V/dec)	β_c_ (V/dec)	i_corr_ (A/cm^2^)	E_corr_ (V) vs. SCE
*S. algae*	0.304	−0.130	2.38 × 10^−5^	−0.997
*S. algae* + IOB	0.202	−0.129	2.20 × 10^−5^	−0.896
IOB	0.196	−0.126	1.81 × 10^−4^	−1.027

**Table 6:** EIS parameters of L245 corroded in different microbial systems for 7 days.

Condition	Day	*R*_s_ (Ω·cm^2^)	*Q*_f_ (F·cm^−2^)	*R*_f_ (Ω·cm^2^)	*Q*_dl_ (F·cm^−2^)	*R*_ct_ (Ω·cm^2^)
*S. algae*	1	2	0.0001	3	0.0002	35,060
	3	2	0.0001	216	0.0002	50,090
	5	2	0.0002	248	0.0002	14,010
	7	2	0.0003	575	0.0002	4,790
*S. algae* + IOB	1	3	0.0007	2	0.0004	354
	3	3	0.0013	921	0.0006	3,753
	5	6	0.0015	3,496	0.0006	10,290
	7	6	0.0014	2,835	0.0004	21,800
IOB	1	2	0.0001	433	0.0002	7,862
	3	3	0.0001	9	0.0002	7,351
	5	2	0.0003	181	0.0002	4,423
	7	2	0.0099	99	0.0428	300

## Data Availability

The original contributions presented in this study are included in the article. Further inquiries can be directed to the corresponding author.
